# Design of a Purely Mechanical Sensor-Controller Integrated System for Walking Assistance on an Ankle-Foot Exoskeleton

**DOI:** 10.3390/s19143196

**Published:** 2019-07-19

**Authors:** Xiangyang Wang, Sheng Guo, Haibo Qu, Majun Song

**Affiliations:** 1 Robotics Research Center, School of Mechanical, Electronic and Control Engineering, Beijing Jiaotong University, Beijing 100044, China; 2 Key Laboratory of Vehicle Advanced Manufacturing, Measuring and Control Technology, Ministry of Education, Beijing Jiaotong University, Beijing 100044, China

**Keywords:** mechanical sensor, self-adaptiveness, ankle-foot exoskeleton, walking assistance

## Abstract

Propulsion during push-off (PO) is a key factor to realize human locomotion. Through the detection of real-time gait stage, assistance could be provided to the human body at the proper time. In most cases, ankle-foot exoskeletons consist of electronic sensors, microprocessors, and actuators. Although these three essential elements contribute to fulfilling the function of the detection, control, and energy injection, they result in a huge system that reduces the wearing comfort. To simplify the sensor-controller system and reduce the mass of the exoskeleton, we designed a smart clutch in this paper, which is a sensor-controller integrated system that comprises a sensing part and an executing part. With a spring functioning as an actuator, the whole exoskeleton system is completely made up of mechanical parts and has no external power source. By controlling the engagement of the actuator based on the signal acquired from the sensing part, the proposed clutch enables the ankle-foot exoskeleton (AFE) to provide additional ankle torque during PO, and allows free rotation of the ankle joint during swing phase, thus reducing the metabolic cost of the human body. There are two striking advantages of the designed clutch. On the one hand, the clutch is lightweight and reliable—it resists the possible shock during walking since there is no circuit connection or power in the system. On the other hand, the detection of gait relies on the contact states between human feet and the ground, so the clutch is universal and does not need to be customized for individuals.

## 1. Introduction

Walking efficiency significantly influences the walking duration and metabolic cost of the human body. Humans have evolved a way of walking under natural selection, which is well-tuned and might be the most energetically efficient [1]. Since half of the positive work is done by the ankle during push-off [2], the design of ankle-foot exoskeleton (AFE) has become a hot research topic in the past years, and many devices have been developed to reduce the metabolic cost when normally walking [3,4,5,6,7]. The assistance or the torque provided by actuators rely on the recognition of people’s real-time gait data and the adjustment of the control law promptly [8,9,10,11]. As a result, many gait-phase detection methods and algorithms have been proposed to provide accurate status feedback on the current gait stage. 

Existing wearable sensor systems can be divided into the following types, including Electromyography (EMG) sensors [12], footswitches [13,14,15], foot pressure insoles [16], joint encoders, and inertial sensors [17]. 

The electrical activity in the gastrocnemius or soleus is an intuitive prediction of movement, which is employed by the control system of many AFEs [18,19,20]. There is no detection delay, and the voltage message can be detected as early as 50 ms before the contraction of muscles [21]. However, the signal varies when physical or physiological changes happen [22], for example, the magnitude changes due to the location shift of the electrodes [23], or the frequency drifts due to muscle fatigue [24]. Some complex processing approaches are also required since the sensing signal is too weak [25]. Force sensors are widely used in the detection of gaits, such as the foot pressure insoles which should be customized for different individuals, and the footswitches that have comparatively higher robustness by setting threshold rules [26], even though the number of the detectable gait phase is limited [27]. Sawicki et al. [28] have designed an AFE by controlling the contraction of the artificial muscle according to the contact states of the forefoot and effectively reduced the metabolic cost during walking.

The inertial sensors and the joint encoders can record and provide the kinematic information on the limb, and the system states of the controller [29,30,31]. However, the signal lags behind the EMG with respect to the generation time [32]. Recently, wearable capacitive sensors [33,34] and force myography-based sensors [35,36] have also been utilized for gait event detection and a satisfactory accuracy is achieved, but they have never been used for AFE control. 

In order to increase the detecting accuracy of the gait phase, multi-sensors are usually integrated to achieve fusion-based recognition [37]. Together with the controller and actuators, a device with the bulk of the electronic control system is worn at the distal limb, resulting in an additional metabolic cost to the human body. It has been shown that the metabolic increase associated with adding mass to the foot is more than four times greater than the same mass attached to the waist [5,38]. The actuator and sensor system must be light so that the benefit from the energy input will not be counteracted by the additional metabolic cost caused by adding mass. Moreover, nearly all the sensors mentioned above are circuit-based. Shock during walking is an adverse factor towards the sensing accuracy and can destroy the circuit. When people walk fast, the delay in signal transmission, processing, and calculation may also arouse some problems. 

Purely mechanical sensors are reliable when compared with the circuit-based sensors mentioned above because the signal transmission procedure relies on the motion of components under mechanical constraints. Also, the forces exerted by the environment can be transmitted by the rigid body without any delay. Collins [39,40] has designed a clutch based on the ratchet-pawl mechanism so that the spring linkage could be controlled by setting the timing of the pawl latch and release, which actually could be regarded as a mechanical sensor that is capable of detecting the key gait event during walking. However, the device must be customized to fit for different gait characteristics. Yandell et al. [41] presented an unpowered ankle exoskeleton with a slider under the shoes. The slider, together with a top and a bottom gripper, can identify a specific gait stage and control the transformation of energy so that the walking efficiency could be hugely improved. 

The purpose of this paper is to design a purely mechanical sensor-controller integrated system to achieve the goal of gait identification and to reduce the metabolic cost during human walking. This system consists of a sensing part and a mechanical executing part. The sensing part acts as a sensor that possesses two input channels, while the executing part works like a controller that controls the engagement of a suspended spring behind the calf muscles. Unlike other electronic sensors that can record and transmit the data to the microprocessor unit for post-processing purposes, the sensing part must work together with the executing part, so that the motion signal could be “read” and contribute to the control of the spring in a mechanical way. Since the system is connected directly to the actuator (spring), in a purely mechanical system, the mechanical sensor should be analyzed together with the controller. That is the main difference between mechanical sensors and the circuit-based electronic sensors. 

We briefly introduce the biomechanics method and goals of the design in Section 2.1 and Section 2.2 and describe the structure and working process of the clutch in Section 2.3 and Section 2.4. In Section 3, we simulate to evaluate the metabolic cost of the plantar muscles when the passive AFE is worn on the human body and conclude in Section 4.

## 2. Methods

### 2.1. Biomechanics and Energetics during Human Walking

**Figure 1:**
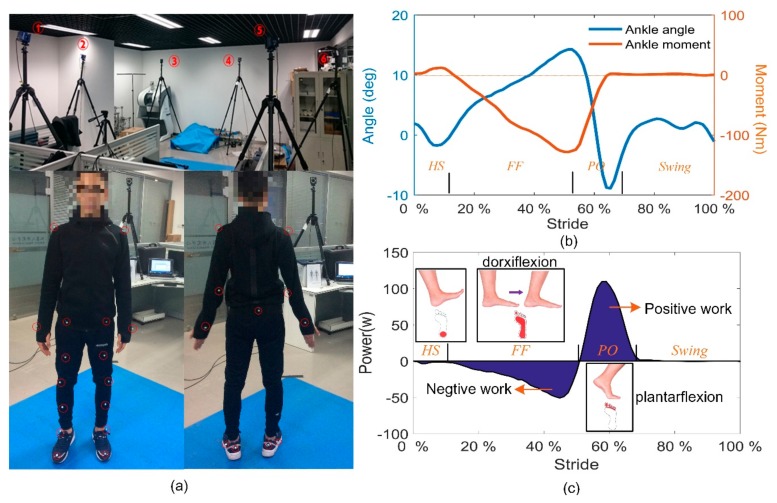
Kinematic data collection during the normal walking process. (**a**) The Helen Hayes marker set placement. (**b**) The obtained ankle angle and moment data within one gait cycle. (**c**) Instantaneous power of the ankle joint within one gait cycle.

In order to study the movement mechanism of the ankle joint and find the nature of energetics during human walking, we carried out a gait experiment. A 3D motion capture system (Cortex, Motion Analysis Co., Santa Rosa, CA, USA) was used to track the body movement. The Helen Hayes marker set was used with 15 markers worn on the lower limbs and six markers on the torso (including arms), as shown in Figure 1a. 

Testers walked through an irradiation area formed by six cameras. The ground reaction force (GRF) was measured during walking along a 2.4 m walkway formed by four force plates (JP4060, Bioforcen Intelligent Tec. Ltd., Hefei, China). The captured gait cycle could be divided into four stages according to the contact state between the shoe sole and the ground. The cycle begins with the heel strike (HS) and experiences Flat foot (FF), Heel off (HO), and the swing phase. The ankle angle and moment were respectively calculated based on the kinematic data and the GRF data in the software cortex (see Figure 1b). As shown in Figure 1c, dorsiflexion happens almost during the FF, whereas plantarflexion is with the process of PO. By multiplying the joint velocity and the ankle moment, the instantaneous power of the ankle joint is derived. The ankle joint output work with completely different properties at different gait stage, i.e., negative work during FF, and positive work during PO. Although both positive and negative work cost energy to the human body [42], the positive work is usually performed to move body segments and increase the potential energy of body center of mass (COM), whereas the negative work is done with the mechanical energy (ME) transferred into other forms of energy. The reason for the negative part could be the energy stored in the tendons, and the energy dissipated due to the damped motion of fat, viscera, and muscle [43], which means only a part of the energy could be recycled, with most of the energy wasted. 

Man consumes his biomass energy continuously to maintain the walking speed and compensate for the energy loss due to physiological activities in his body. Since energy is recycled with an elastic component almost without any loss, the increasing proportion of the energy transition into the elastic components could greatly reduce the energy consumed by the human tissue, increasing the walking efficiency, and achieving better walking economy.

### 2.2. Bio-Inspired Passive AFE and Its Description

**Figure 2:**
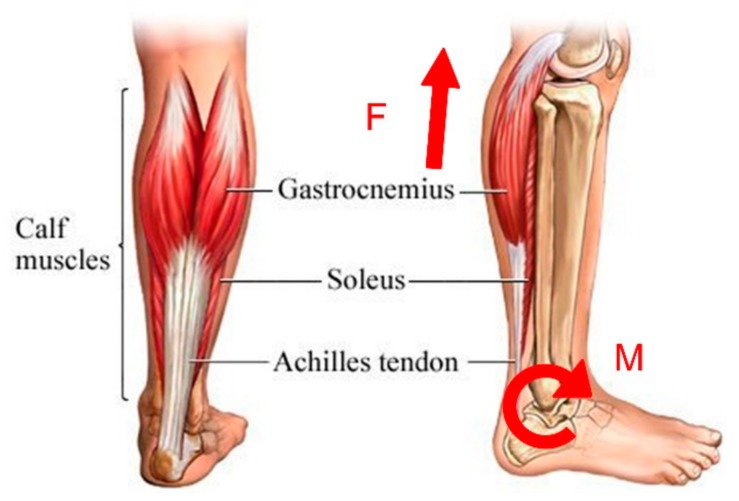
The composition of the calf muscles, which plays a key role in propelling human body forward [44].

Tendon-muscle units play an important role in the realization of human movements. Soleus and gastrocnemius (see Figure 2) are the most important plantar muscles. When a person begins to raise his heel, the calf muscles contract and generate a linear force F that causes a clockwise moment M about the rotation center of the ankle joint, outputting quantities of mechanical work.

**Figure 3:**
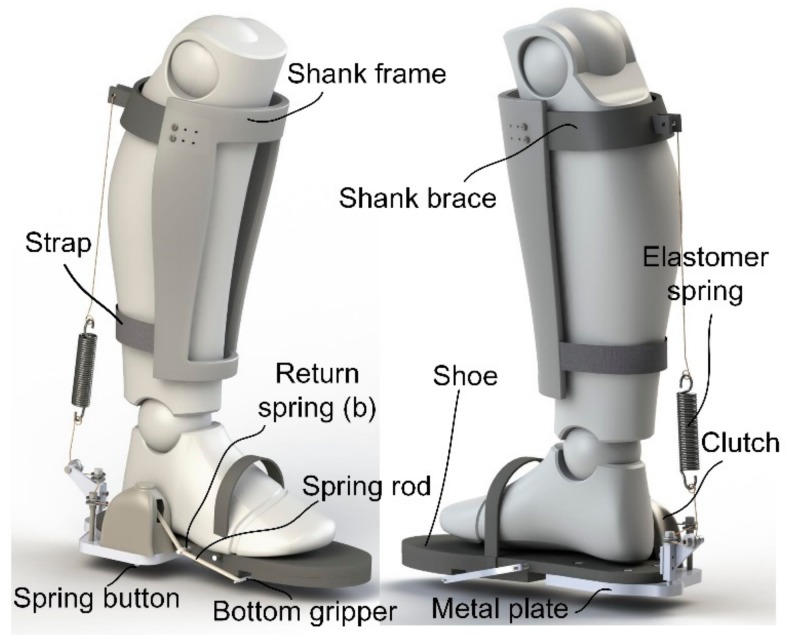
The proposed passive ankle-foot exoskeleton (AFE) and its components. The core and fundamental part is the clever clutch that ensures the implementation of the function.

Inspired by this, we developed a passive AFE that has a structure similar to a tendon-muscle unit (see Figure 3). An elastomer spring is suspended behind the calf muscles aiming to generate a linear force parallel to the muscle group during plantarflexion. The spring should be stretched at the beginning of FF, storing energy during the pendular motion of the human body, and transfers the energy into strain energy in the process of dorsiflexion. At the end of FF, the stored energy reaches its maximum and could be recycled to provide assistance. During the swing phase, the suspended spring is supposed to be disengaged, without impeding the free movement of the ankle joint. 

In order to achieve the goal proposed above, a clever sensor-controller integrated system was designed to identify the key event of gait and control the engagement of the spring. A shoe is cut off from its back, with the upper heel kept to serve as a base, to which a metal plate is connected, so that the wearer could keep their foot completely flat when normally standing. The clutch is placed on the metal plate. 

The interface between the shank and the exoskeleton consists of a shank brace and a shank frame. Since the tension of the spring can be up to hundreds of newtons, the possible slipping of the shank brace should be avoided by adjusting the position of the shank frame according to the size of the user’s shank. With a nylon strap attached to the lower end of the frame, the spring force is held by the whole shank frame, so that comfort during its use is guaranteed. 

### 2.3. Sensor-Controller Integrated System and Its Design

#### 2.3.1. Mechanical Structure of the Clutch

The clutch is the core component in the design of the passive AFE that ensures that self-adaptiveness can be achieved. Self-adaptiveness here is defined as the capability of identifying different gait stages and providing assistance at a proper time mechanically. 

**Figure 4:**
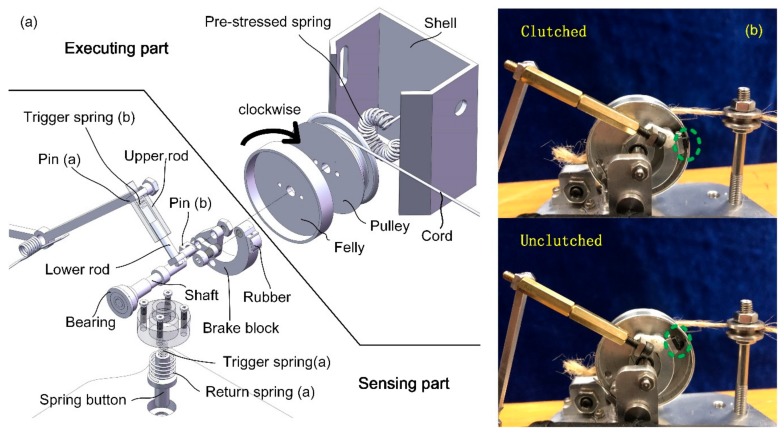
(**a**) The structure of the mechanical clutch. This sensor-controller integrated system consists of a sensing part and an executing part; (**b**) The clutched/unclutched state of the prototype, which relies on the contact state between the rubber and the wall of the felly flange.

Fixed at the outer side of the shoe sole, the clutch is composed of several tiny components. The overall size is small enough to be put into a spherical ball with a diameter of 42 mm. Figure 4a shows the mechanical structure of the clutch in a zoomed view. The whole clutch is divided into two parts, namely, the sensing part and the executing part. The executing part comprises a felly, a pulley, a pre-stressed spring, and a cord with one end attached to the pulley. The remaining components inside the clutch constitute the sensing part. The felly and the pulley are bolted together, concentric with a shaft passing through their center holes. Hence, the cord can be dragged out when the felly rotates clockwise (CW) and is dragged in when the felly rotates in the opposite direction. Routed to the back of the foot, the other end of the cord passes through three bearings and is connected to the lower end of the elastomer spring, the upper end of which is fixed at the shank brace. When the clutch is clutched (see Figure 4b), the rotation of the felly is restricted. As a result, the elastomer spring can only be stretched during the dorsiflexion process with elastic energy accumulated. 

**Figure 5:**
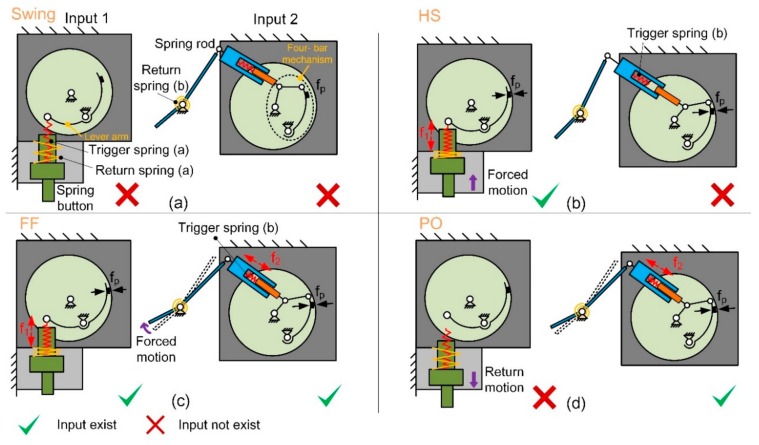
State of the executing part and the corresponding gait phases. (**a**) Swing: neither of trigger springs (a) and (b) is compressed; (**b**) heel strike (HS): only trigger spring (a) is compressed; (**c**) flat foot (FF): both trigger springs (a) and (b) are compressed; (**d**) push-off (PO): only trigger spring (b) is compressed.

In order to control the states switching of the clutch, a spring rod and a spring button are separately distributed at the fore shoe sole and the rear sole. They are pushed under the GRF. The forced motion of the spring button results in the compression of the trigger spring (a) (see Figure 5a) so that one end of the lever arm moves upward under the spring force *f*_1_ (see Figure 5b), which results in the contact between the rubber and the flange of the felly with normal pressure *f*_*p*_, causing a friction force. Similarly, when the spring rod is pushed under GRF, the trigger spring (b) is compressed (see Figure 5c). The spring force $f_{2}$ is generated and transmitted by a four-bar mechanism, pushing the rubber to contact the flange of the felly (see Figure 5d).

**Figure 6:**
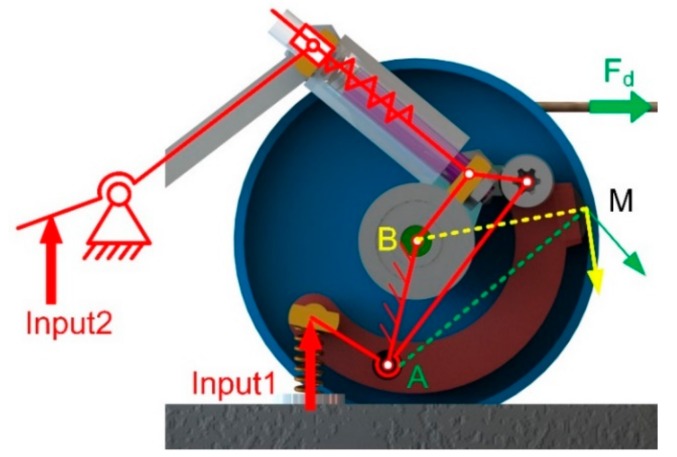
Two-inputs mechanical sensing system. For Input 1, the force is transmitted by a leveling mechanism. For Input 2, the force is transmitted by a four-bar mechanism.

When the rubber contacts the felly, the clutch is clutched due to the mechanical constraints. As shown in Figure 6, point M has different velocities with points A and B as the center of rotation. There is no doubt that point M cannot move along two directions at the same time. The pulling force *F*_*d*_ will cause deformation of the rubber, resulting in a larger normal pressure $f_{p}$ and a corresponding friction force that stops the CW rotation of the felly. The greater the pulling force on the cord, the greater the resistance to rotation. Consequently, the clockwise rotation of the pulley is completely restricted.

**Figure 7:**
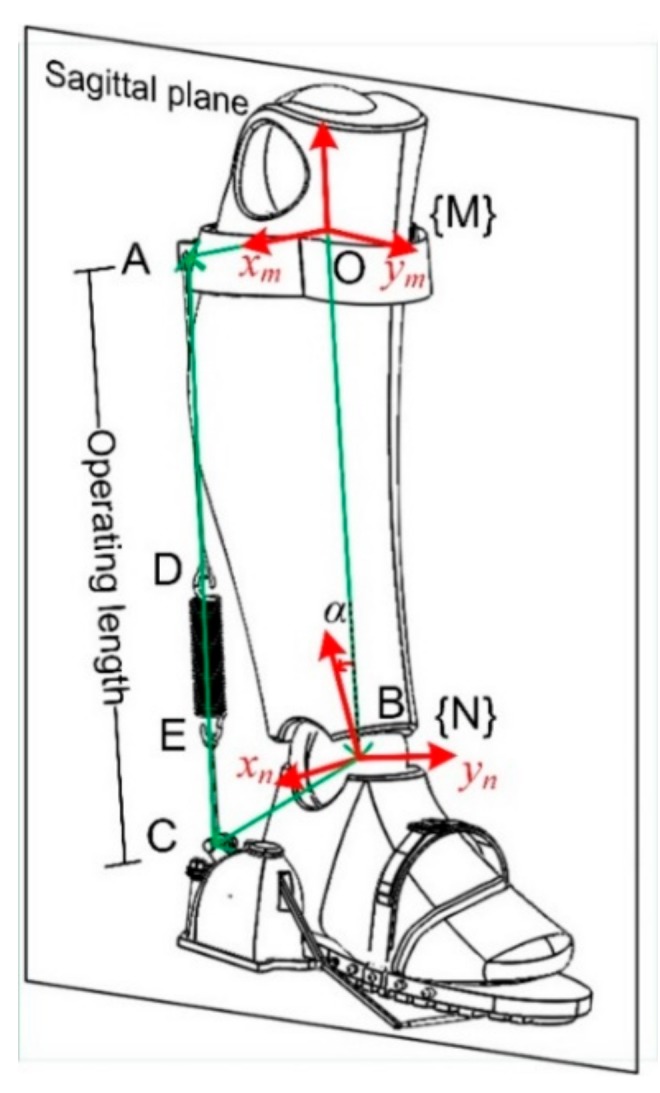
The sagittal plane and the definition of the operating length. $x_{n}$ and ***x***_*m*_ are all perpendicular to the sagittal plane.

In order to keep the cord taut all the time, a pre-stressed spring is placed inside the pulley with one of its ends fixed to the shell and the other end attached to the pulley. The pre-stressed spring could produce a counterclockwise torque. When the cord is slack, or the distance between point A and C is reduced (A and C are defined in Figure 7), excess cord length is dragged into the pulley immediately. Consequently, the cord always keeps tensioned.

Since the friction moment caused by the contact force *f*_*p*_ decreases as the felly counterclockwise (CCW) rotates, CCW rotation could still be achieved under the action of the pre-stressed spring. During the swing phase, the brake block leaves the flange due to the auto-return characteristics of the spring-linkage structure so that the clutch is unclutched. Two-way rotation is, therefore, allowed with the elastomer spring disengaged, which guarantees the full range movement of the ankle joint.

Each trigger spring is an independent input signal that controls the position of the brake block and the rubber attached to it. The state of the clutch under different conditions is shown in Table 1.

It could be concluded that when at least one input exists, the executing part is clutched and only one-way rotation of the pulley is allowed, which means the cord could not be dragged out from the pulley as the foot dorsiflexes. When no inputs exist, the clutch is unclutched, and two-way rotation is allowed, without impeding the free movement of the ankle joint. The return springs (a) and (b) ensure the spring button and the spring rod reverts to their original state when there is no GRF.

#### 2.3.2. Working Principle

In the design of the passive AFE, the increment of the operating length corresponds to the extent of the energy stored. In the sagittal plane, the length could be derived through kinematic calculations. 

The coordinates systems {M} and {N} are attached to the shank and the foot respectively, with *z*_*m*_ pointing along BO (B is the center of the ankle joint, O is attached to the shank as shown in Figure 7). {N} is obtained by rthe otation about *x*_*m*_ with an angle α, as shown in Figure 7, a vector loop equation is obtained

(1) CA=OA−OB−BC

Solving Equation (1) for CA, we obtain

(2) CA=OAxOAyOAz−00−l−RNMBCxBCyBCz

And the coordinate-transformation matrix from coordinate {M} to {N} is

(3) RNM=1000cosα−sinα0sinαcosα

where, angle α is obtained via inverse kinematics based on movement information collected by the motion capture system. 

**Figure 8:**
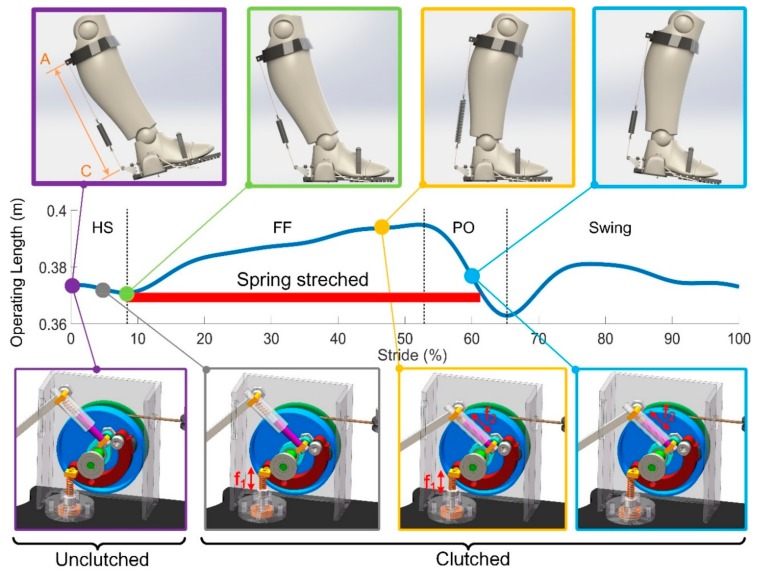
Schematics showing the working mode with respect to stride and the key event of the clever clutch in different gait phases.

The operating length ranges from 370 mm to 395 mm in one gait cycle when normally worn, which means the spring could be stretched up to 25 mm with the corresponding energy stored. The maximum moment arm is about 98.2 mm. The states of the clutch and the contact status between the shoe and the ground, together with the variation of the operating length are presented in Figure 8.

In the beginning, the human foot is in the swing phase with the elastomer spring disengaged (in purple). The clutch allows the two-way rotation of the pulley without hindrance applied to the ankle joint. When the spring button is moved under GRF during HS, the operating length decreases a little bit as the ankle angle decreases, with the excess cord immediately pulled into the pulley by the pre-stressed spring (in grey). At maximum plantarflexion (in green), the foot is completely on the ground with the operating length reaching its minimum and preparing to perk up. Both the spring rod and the spring button are simultaneously pushed, resulting in the clutched state of the executing part until the end of the FF, which prevents the cord from being pulled out as the shank rotates. As a result, the elastomer spring is engaged and stretched as the ankle begins to dorsiflex into the terminal stance (in yellow).

The spring rod keeps the clutch clutched as the heel of the sole is raised with the spring button coming back to its original position (in blue), which ensures the steady release of the energy is achieved without any loss. The required force from the muscles is hugely reduced with the assistance provided by the spring.

Following the push-off, the clutch switches to be unclutched as the foot is off the ground.

#### 2.3.3. Clutch Prototype Specification and Testing

The implementation of the state switching of the clutch significantly affects the timing when the assistance should be provided and ensures that free rotation of the ankle is achieved at the interval when no assistance is in need. In order to test the reliability of the switching states, a prototype is produced and tested.

**Figure 9:**
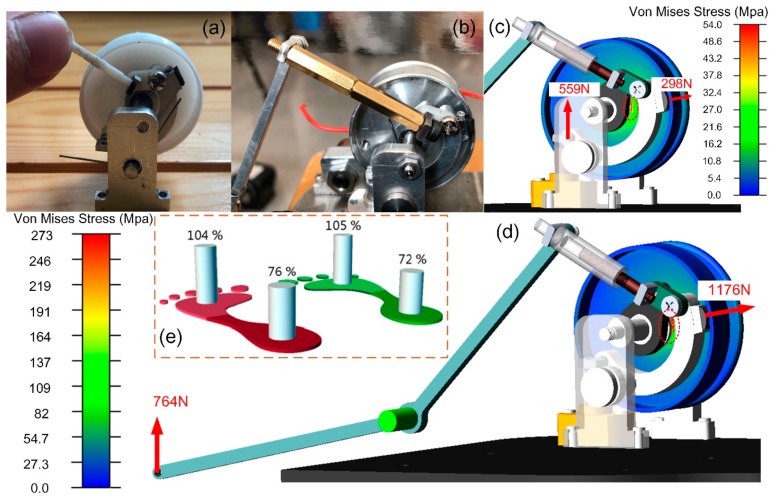
The prototype of the proposed sensor-control integrated clutch. (**a**) Original prototype with 3D printed pulley, felly, and connecting rod; (**b**) Second prototype with all components made of Al-alloy. (**c**) Finite element model when the bottom gripper suffers from the ground reaction force (GRF); (**d**) The finite element model when the bottom gripper suffers from the GRF; (**e**) The distribution of the body weight at the fore and rear foot.

In our prototype, the felly may be deformed under normal pressure *f*_*p*_, which is mainly affected by two factors, namely, the pulling force on the cord and the GRF transmitted by the two mechanical inputs. Too high normal pressure could lead to mechanical failure. Considering the mass of the device, we first used 3D printed material (ABS) to build our prototype. For the convenience of the strength test, we used two shaft seats to hold the clutch mechanism. A torsional spring is placed on the shaft with its two arms attached to the brake block and the shaft seat respectively, ensuring that the clutch was unclutched when no inputs trigger the clutch. The flange wall has a thickness of 1 mm with a textured surface. When the cord is stretched and the clutch is set to be the clutched state, the resistance increases as the felly slightly rotates. The rubber is compressed as the pulling force increases, and the larger the pulling force, the larger the normal pressure $f_{p}$ on the contacting area between the rubber and the wall of the felly flange, thus generating a larger friction force to stop the rotation of the felly. When the spring force was larger than 35N, the felly generated a visible deformation due to the characteristic of the plastic material. In practice, the pulling force could be up to a few hundred newtons when stiff spring is used. Hence, metal should be used instead. To achieve a better mass stiffness ratio, Al-alloy is employed as the base material in our test, as shown in Figure 9b. In our testing with the second prototype, the felly remains undeformed after a pull is exerted. 

Assuming that the components in the two mechanical channels are all rigidly connected, which happens as the trigger spring (a) and (b) are fully compressed. GRF causes larger pressure than that caused by pulling the cord. In order to evaluate the structural limits of our prototype with the material Al-alloy, we carried out a finite element analysis based on the software Adams. When a person normally walks, the distribution of the pressure at the fore sole and the rear sole could be measured. In our case, one of the human subjects (24 years old, 180 cm tall, 74.2 kg) walked on a treadmill (Zebris FDM-T, Zebris Medical GmbH, Isny, Germany) at a speed of 4 km/h, the maximum load (% of body load) at the forefoot and the rear foot were respectively recorded, as shown in Figure 9e. The result showed that the forefoot carries 105% of the body weight, whereas the heel takes up only 76%, which implies the maximum GRF applied to the bottom grimmer and the spring rod is 559 N and 764 N, respectively.

Based on the above loading conditions, the pulley was meshed into a tetrahedral shape with the average element size of 0.2 mm in the simulation environment. The coefficient of friction between the rubber and the Al-alloy is 0.4. Assuming all the parts are rigid bodies except for the felly, we added the force directly to the pin (b) or the bottom gripper independently to compare the stress results. 

The results are obtained and shown in Figure 9c,d. In both cases, the stress concentration points are mainly distributed near the contacting line between the felly and the bearing on the shaft (a). For case (b), the maximum Von Mises Stress is about 54 Mpa. As for the case (d), the maximum stress is approximately 273 Mpa. Since the elastic limiting pressure of the 6061-T6 Al-alloy is 333.75 Mpa in room temperature, the clutched clutch will generate invisible deformation during normal walking when the body weight of the users is less than 90 kg (corresponding to the elastic limit). The initial friction force ranges from 120 N to 470 N. When the felly clockwise rotates under the action of the pulling force on the wire rope, the normal pressure increases rapidly when the rubber block is deformed and compressed. Due to the mechanical constraint, the increased friction force prevents the pulley from being rotated and ensures the clutch is completely clutched.

### 2.4. Performance Evaluation

Unlike other circuit-based electronic sensors usually evaluated by the comparison between the detection results and the actual value, mechanical sensors only provide state feedback in the form of partial motion that can be directly “read” by the controller and the actuators. Hence, it works better to focus on the evaluation of the performance of the whole system. 

**Figure 10:**
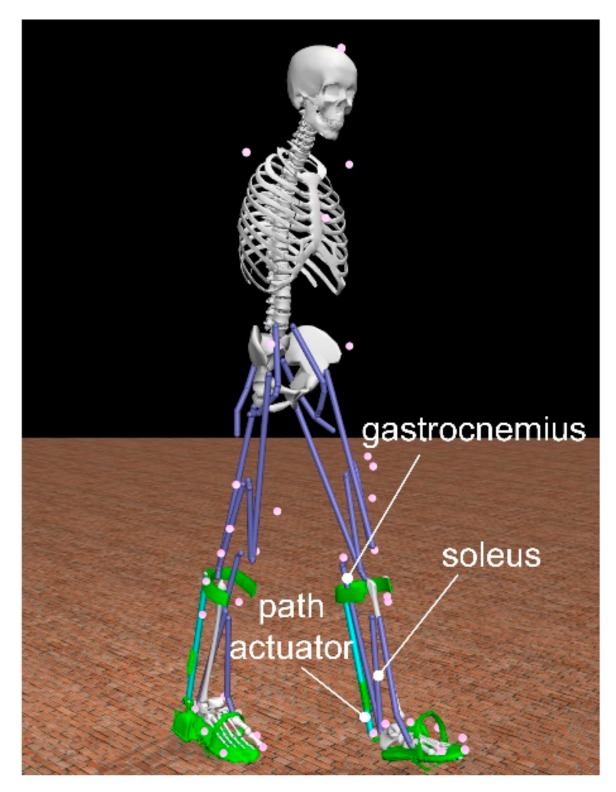
A Human musculoskeletal model with the passive AFE worn on both legs in the environment in OpenSim.

With the help of the software OpenSim [45], we carried out a musculoskeletal simulation with a human skeleton model wearing the AFE. The model weights 72 kg and is 1.8 m tall (see Figure 10). In our case, the stance phase lasts 0.8 s. Since the assistance is provided only during the PO, the stance phase is selected as the main focus. Based on the force-displacement relation of spring, we defined two path actuators to mimic the force output of the elastomer spring. A set of muscle forces that drive a dynamic musculoskeletal model to track the walking behavior has been calculated by the method of Thelen [46].

## 3. Simulation Results and Discussion

**Figure 11:**
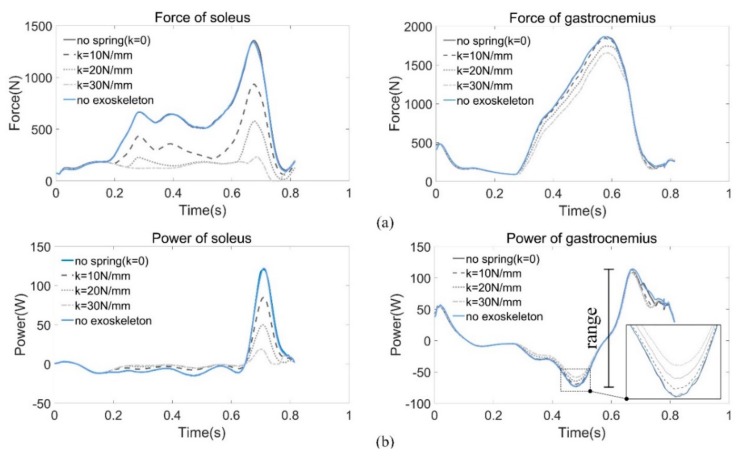
(**a**) Forces of soleus and gastrocnemius under different stiffness conditions. (**b**) Power comparison during the stance phase.

As shown in Figure 11, we compared the forces and power of the plantar flexor muscles (i.e., gastrocnemius and soleus) of the right leg by adjusting the spring stiffness, because these two muscles contribute a major part of the positive work during PO to propel human body forward. 

Data of the force and power are listed in Table 2. The range of power is defined as:

(4) $$P_{r}=P_{\max}-P_{\min}$$

As the stiffness increases, the required muscle forces decrease consequently, especially for the soleus. For every 10 N/mm increase in stiffness, the force decreases nearly by one third, as does the change in power. The changes in gastrocnemius force and power are not as obvious as soleus, but the decline is also noticeable. It is worth noting that there is little difference between the case without assistance (*k* = 0 N/mm) and the case of normal walking since the weight of the exoskeleton is directly transferred to the ground during the stance phase. Therefore, there is almost no effect on the muscles. 

**Figure 12:**
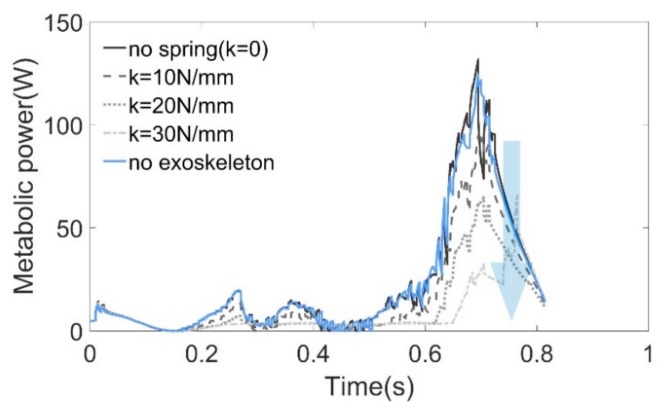
Metabolic cost of soleus under different stiffness conditions.

Metabolic probes were used in the simulation to evaluate the metabolic consuming based on the model proposed by Umberger [47,48]. Since the whole-body metabolic rate is difficult to obtain by simulation, we then estimated the metabolism of the soleus muscle as an alternative. As shown in Figure 12, the results obtained from the probes have almost the same tendency and magnitude as the work rate obtained by computing the dot product of the muscle force vector and the contraction velocity (see Figure 11b). It is difficult to attribute changes in whole-body metabolic rate to a particular change of muscle mechanics [49]; however, simply reducing muscle force can save metabolic energy [39].

**Figure 13:**
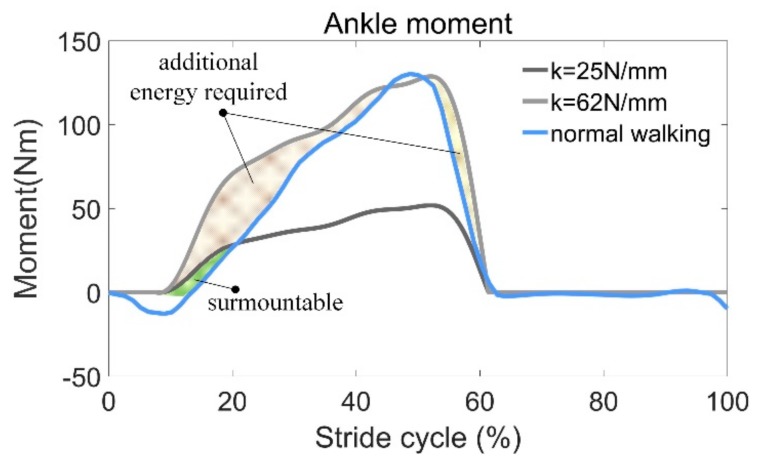
Comparison between the required joint moment and the calculated moment contributed by the AFE.

In order to facilitate the design, an upper limit for the stiffness (*k* = 62 N/mm) is found where the peak ankle moment during normal walking can be achieved. The moment provided by the AFE is then calculated and compared with the actual ankle moment of the musculoskeletal model (see Figure 13). It can be seen that during most of the stance phase (0–60% stride cycle), the AFE moment is greater than the required value. As a result, muscles antagonizing soleus must output additional positive work, so that the desired joint trajectories can be realized. Additional energy must be provided by the human body to compensate for the energy required to be stored in the spring as it is stretched. This can leads to wearing discomfort and even hinder the normal movement of the human body. In addition, 62 N/mm is an incredibly stiff spring and hard to be manufactured.

Even within a certain range, the higher the stiffness, the more energy can be recycled, but some tradeoffs must be made. Due to the individual weight differences, there is no suitable spring stiffness. On the one hand, excessive stiffness may change one’s walking habit; on the other hand, it is possibly slower and could even hinder the pendular motion of the human body. The stiffness *k* = 25 N/mm is a recommended value based on the simulation results (see Figure 11a), where the soleus force approaches to zero during push-off when k=20 N/mm and decreases to zero when *k* = 30 N/mm. Hence, we took an intermediate value. Since the exoskeleton plays a role in assisting, rather than a role that replaces the human muscles, we hoped the force of soleus was larger than zero all the time. 

In our case, the maximum moment arm was 98.2 mm, and the maximum moment provided by AFE was 52.4 Nm. Compared with the peak value of the torque during normal walking (130.3 Nm), only 59.8% of the moment is required from the human body. Even additional energy is still required at the beginning of FF (12–50%), but this can be overcome easily without too many hindrances applied to the human body. 

## 4. Conclusions

Based on the analysis of the movement mechanisms and energetics in different gait phases, we designed a novel passive AFE in analogy with the muscle-tendon unit to mimic the muscle force. Such a device is capable of storing energy and providing assistance during human walking. In order to detect the gait phases mechanically, a sensor-controller integrated system, or the clutch, was designed and introduced. The sensing part has two-inputs that are used to detect the contact status between the shoe sole and the ground, and each of the inputs could control the state of the executing part independently. 

The energy storage and release process rely on the state of the clutch. When the clutch is clutched, the spring suspended behind the calf muscles is engaged. The energy is stored in the process of plantarflexion and released during the PO to provide walking assistance. When the clutch is unclutched, the spring is disengaged, without impeding the free rotation of the ankle joint.

Since the mechanical sensor is incorporated into the system, the assistance of the AFE under the control of the clutch is evaluated by the simulation. By comparing the force and power of plantar muscles under different stiffness conditions, the relationship between the spring stiffness and the assistance provided is obtained. The metabolic cost of the soleus is also estimated to show the walking economy.

The system is entirely passive and user-friendly with high reliability and can resist the disturbance of shocking during human walking. In our future work, we plan to make a prototype and do a more related test with the AFE worn on the human body.

## Figures and Tables

**Table 1:** State of the clutch during different gait phases.

Gait Phase	Forced Motion (Spring Button)	Forced Motion (Spring Rod)	State of the Executing Part	Rotation Restricted
Swing	No	No	unclutched	-
HS	Yes	No	clutched	CW
FF	Yes	Yes	clutched	CW
PO	No	Yes	clutched	CW

**Table 2:** Comparison of forces and power of plantar flexor muscles.

Reduction (in brackets)	Soleus		Gastrocnemius	
	Maximum Force (N)	Range of Power (W)	Maximum Force (N)	Range of Power (W)
no exos	1341.8	122.0	1865.1	186.8
no spring	1356.2 (−1.1%)	120.6 (−1.1%)	1866.4 (−0.07%)	187.42 (−0.33%)
10 N/mm	934.3 (30.4%)	84.9 (30.4%)	1844.9 (1.1%)	180.7 (3.3%)
20 N/mm	578.3 (56.9%)	50.0 (59.0%)	1745.9 (6.4%)	173.2 (7.3%)
30 N/mm	234.2 (82.5%)	18.8 (84.6%)	1657.7 (11.12%)	172.4 (7.7%)
